# Arterial stiffness in the context of heart rate and vasomotion during orthostatic stress and cognitive load

**DOI:** 10.3389/fnetp.2026.1829189

**Published:** 2026-06-24

**Authors:** Barbora Czippelova, Zuzana Turianikova, Jana Cernanova Krohova, Nikoleta Mazgutova, Michal Javorka

**Affiliations:** Department of Physiology and Biomedical Centre Martin (BioMed Martin), Jessenius Faculty of Medicine in Martin, Comenius University in Bratislava, Martin, Slovakia

**Keywords:** network physiology, arterial stiffness cardio-ankle vascular index (CAVI0), orthostatic stress, cognitive load, blood pressure, heart rate, systemic vascular resistance

## Abstract

**Introduction:**

Variability in cardiovascular parameters reflects complex interactions among regulatory mechanisms. Arterial stiffness, a key determinant of heart–vessel interaction and cardiac performance, is commonly assessed using pulse wave velocity, though its dependence on blood pressure limits interpretability. The Cardio-Ankle Vascular Index (CAVI), a widely used non-invasive measure of arterial stiffness, and its refined form CAVI_0_, aim to address this limitation. However, evidence suggests that these measurements may still be affected by factors such as vascular smooth muscle tone and heart rate. This study investigated the short-term effects of physiological stressors, including body position changes (considering hydrostatic pressure) and cognitive load on CAVI_0_, to better understand the influence of confounding factors on arterial stiffness assessment.

**Methods:**

Twenty-three healthy adults (age 22.3 ± 2.4 years, 16 male) were recruited. CAVI_0_ was measured using the VaSera VS-1500, with adjustments for blood pressure and hydrostatic effects (CAVI_0__ADJ_) during three randomized tilt sequences (10° head-down tilt (HDT), 20° head-up tilt (HUT), and 40° HUT) and a mental arithmetic (MA) task inducing cognitive load. Continuous ECG, volume clamp photoplethysmography, and impedance cardiography recordings were used to derive heart rate, blood pressure, cardiac output, and systemic vascular resistance.

**Results:**

CAVI_0__ADJ_ increased progressively, showing significant elevations at 20° and 40° HUT versus supine and 10° HDT. Brachial mean blood pressure did not change across postural positions, whereas heart rate and systemic vascular resistance increased during upright postures. Changes in CAVI_0__ADJ_ positively correlated with heart rate (p < 0.001) and systemic vascular resistance (p = 0.040), but not with brachial mean blood pressure. During the MA task, brachial mean blood pressure, heart rate, and systemic vascular resistance all increased significantly, whereas CAVI_0_ remained unchanged.

**Conclusion:**

Our study shows that CAVI_0_ is not influenced by acute blood pressure changes caused by moderate mental stress pointing towards its robustness against blood pressure alterations. During head-up tilt, CAVI_0_ is influenced not only by hydrostatic pressure but also by heart rate and vasomotion, highlighting the importance of considering body position and autonomic responses when assessing arterial stiffness as an indicator of structural changes in arterial wall associated with atherosclerotic process.

## 1 Introduction

Cardiovascular system is characterized by many parameters–nodes in the network–mutually interconnected by complex mechanical and reflex connections resulting in their rich dynamics (Bashan et al., 2012; Ivanov, 2021). Cardiovascular parameters continuously oscillate as a result of complex, interconnected regulatory mechanisms. Short term changes in them are mediated either by mechanical connection between variables or by autonomic nervous system. Although cardiovascular regulation is commonly studied using time-series analysis of spontaneous beat-to-beat oscillations, evaluating state-to-state changes in cardiovascular variables in the context of interconnected physiological networks and potential confounders is equally important. Recent research has increasingly focused on interactions among physiological systems rather than isolated cardiovascular variables to achieve a more comprehensive understanding of cardiovascular regulation and improve parameter interpretation (Pichot et al., 2024; Cairo et al., 2023; De Abreu et al., 2022). To further elucidate the mechanisms underlying changes in cardiovascular parameters, it is necessary to analyze not only traditionally evaluated measures (e.g., heart rate (HR), systolic blood pressure (SBP)), but also to more thoroughly characterize the dynamics of additional variables that substantially influence cardiac performance.

Arterial stiffness (or its reciprocal value–arterial compliance) is a very important characteristics of the vascular system significantly influencing heart–vessels interaction and pumping function of the heart. Elevated arterial stiffness is an early indicator of structural changes in the arterial wall—often associated with atherosclerosis—and a key factor in the pathogenesis of systolic arterial hypertension (Kass, 2002). In clinical practice, arterial stiffness is not measured directly. Among the various surrogate measures of arterial stiffness, pulse wave velocity (PWV) is the most widely used, with carotid-femoral PWV (cfPWV) recognized as the reference “gold standard.” Numerous studies have demonstrated a strong association between cfPWV and the presence of coronary, cerebral, and carotid artery atherosclerosis, establishing cfPWV as a valuable tool for diagnosing and assessing the risk of cardiovascular diseases (Kim and Kim, 2019). However, a key limitation of PWV measurement is its dependence on current blood pressure. This drawback has been addressed by the development of the Cardio-Ankle Vascular Index (CAVI) (Shirai et al., 2006). Although CAVI has been considered a pressure-independent index of arterial stiffness, Spronck et al. (2017) challenged this assumption, introducing a modified version known as CAVI_0_.

When interpreting arterial stiffness indices in clinical practice or research, it is important to consider several potential confounding factors that may influence the estimated values. Although CAVI_0_ eliminates the major confounder—blood pressure—other variables may still affect its interpretation and accuracy. These include the tone of smooth muscle cells in large arteries modulated by sympathetic nerve activity (Bank et al., 1995; Czippelova et al., 2019; Nardone et al., 2018), as well as heart rate, which has also been shown to influence arterial stiffness measurements (Lantelme et al., 2002; Tan et al., 2016). A better understanding of changes in CAVI_0_ related to aforementioned confounding factors under conditions that do not meet the standard measurement criteria (e.g., head-down tilt (HDT), head-up tilt (HUT), cognitive load) could provide valuable insight into its short-term variability and potential influence of confounders.

Several studies have examined the influence of body position on arterial stiffness parameters (Pucci et al., 2020; Schroeder et al., 2017; Shimawaki and Yusa, 2016; Maliha and Townsend, 2017). However, these investigations have either assessed pulse wave velocity (PWV) without accounting for blood pressure, or used CAVI adjusted only for brachial blood pressure, without considering hydrostatic pressure gradients in non-supine positions. In the present study, we used CAVI_0_ adjusted for the blood pressure acting along the measured arterial segment, incorporating the contribution of hydrostatic pressure. This approach was intended to minimize the effect of blood pressure on arterial stiffness assessment and to better isolate the contribution of other potential confounding factors. Furthermore, the effect of another stressor—cognitive load—was assessed.

Understanding potential short-term alternations in arterial stiffness–such as those occurring during changes in body position or cognitive load–is crucial for the appropriate interpretation of CAVI_0_ as a marker of clinically meaningful vascular stiffening, particularly in the context of non-invasive monitoring of atherosclerosis progression. Therefore, the aim of the present study was to investigate the effect of these physiological stressors on CAVI_0_, in the context of changes in potential confounding factors. This work thus has the potential to advance the understanding of stress-induced network-level changes in cardiovascular regulation and highlights the importance of a network physiology-based approach to the interpretation of cardiovascular parameter responses.

## 2 Materials and methods

Twenty-three healthy volunteers (mean age: 22.3 ± 2.4 years) were recruited for participation in the study. Current health status was checked by the experienced physician to exclude subjects meeting exclusion criteria. Participants were excluded if they presented with any current infectious disease (including within 3 weeks post-recovery), cardiovascular disease (including hypertension), diabetes mellitus, psychiatric disorders, or hypothyroidism. Anthropometric measurements were obtained using the InBody J10 device (InBody, South Korea). Office blood pressure was measured three times using the auscultatory method during the initial interview. The average of the second and third measurements was used to define each participant’s office systolic and diastolic blood pressure. All participants were classified as normotensive, with office blood pressure values below 140/90 mmHg. Detailed characteristics of the study group are presented in Table 1.

**TABLE 1:** Selected characteristics of participants.

Age (years)	22.3 ± 2.4
Sex (M/F)	16/7
Height (cm)	177.9 ± 10.0
Weight (kg)	73.2 ± 13.5
BMI (kg·m^-2^)	*22*.*3 [21–23.7]*
Fat percentage (%)	*14.4 [11.9–24.2]*
Fat mass (kg)	*10.7 [9.5–15.3]*
Skeletal muscle mass (kg)	*35.6 [27.7–40.3]*
VFA (cm^2^)	*45.3 [40.4–59.1]*
WHR (−)	0.79 ± 0.05
Office SBP (mmHg)	126.2 ± 11.1
Office DBP (mmHg)	83.9 ± 6.0

BMI, body mass index; VFA, Visceral Fat Area; WHR, Waist-to-Hip Ratio; office SBP/DBP, systolic and diastolic blood pressure (mean of 2^nd^ and 3^rd^ sphygmomanometric office blood pressure measurements); data are presented as mean ± SD (normally distributed variables) or *median [interquartile range]* (non-normally distributed variables).

Participants were instructed to abstain from substances known to influence cardiovascular or autonomic nervous system activity (e.g., caffeine, alcohol, energy drinks) and to avoid strenuous physical activity for at least 24 h prior to testing. All measurements were conducted in the morning hours (8:00–11:00 AM) in a quiet, thermoneutral environment (22 °C–25 °C).

All participants provided written informed consent prior to examination and were informed of their right to withdraw this consent any time during or after the examination. The study was approved by the Ethics Committee of the Jessenius Faculty of Medicine, Comenius University (No. EK 44/2018, approved on 24 April 2018), and conducted in accordance with the ethical principles outlined in the 1975 Declaration of Helsinki.

### 2.1 Measurement protocol

The study protocol consisted of several sequential phases. Participants initially rested in the supine position (0°) for approximately 7 min, after which CAVI was measured (measurement duration approximately 1 min). Subsequently, body position was adjusted to one of three tilt angles (10° HDT, 20° HUT, or 40° HUT) using a motor-driven tilt table (Modell 90-00 E, K.H. Dewert GmbH, Germany). These tilt angles were selected due to safety reasons and the technical limitations of our tilt table (our tilt table could not accommodate more negative inclinations). In addition, a more vertical head-up position carried an increased risk of orthostatic intolerance potentially leading to premature termination of the study. Participants remained in the tilted position for 7 min–at the end of this period CAVI was measured again. Thereafter, they were returned to the supine position for a 7-min recovery period to allow physiological parameters to return to baseline. This procedure was repeated for two additional tilt angles, each followed by a supine recovery phase. The order of the tilt angles was randomized across participants. The final part of the examination protocol involved a cognitive load–non-verbal mental arithmetic (MA) task performed in the supine position, lasting approximately 8 min. During the non-verbal MA task, participants were instructed to repeatedly sum the digits of a randomly generated three-digit number until a single-digit result was obtained, then indicate whether the final number was odd or even using a wireless mouse. The attention of participants was distracted by a rhythmic sound of metronome played throughout the task. Participants were instructed to perform the calculations silently without speaking, as quickly and accurately as possible. CAVI measurements were obtained in the first, third, and fifth minutes of the MA task. The first-minute CAVI value was selected for analysis, as peak cardiovascular effects of the mental arithmetic task occur within the first few minutes (approx. 2.5 min) (Lackner et al., 2010).

### 2.2 Arterial stiffness parameter CAVI_0_

A detailed description of the CAVI_0_ methodology and its calculation is available in (Spronck et al., 2017). In brief, the determination of CAVI_0_ is based on the measurement of heart-to-ankle pulse wave velocity (haPWV) along with the diastolic blood pressure. haPWV is obtained by dividing the vascular path length L (estimated from the participant’s height (Takahashi et al., 2019)) by the transit time of the pulse wave from the aortic valve to the ankle. CAVI_0_ is then calculated using the following equation:

$$\text{CAV}I_{0}=\frac{2\rho \cdot \text{haPW}V^{2}}{\text{DBP}}‐\ln\frac{\text{DBP}}{P_{\text{ref}}}$$

where: CAVI_0_ [-] – corrected cardio-ankle vascular index, haPWV [m·s^-1^] – heart-to-ankle pulse wave velocity, ρ [kg·m^-3^] – blood density (1,060 kg.m^-3^), DBP [mmHg] – diastolic blood pressure, P_ref_ [mmHg] – reference blood pressure (P_ref_ = 100 mmHg).

In the present study, CAVI_0_ was obtained by first measuring CAVI with the VaSera VS-1500 system (Fukuda Denshi, Japan), after which the CAVI values were mathematically adjusted to eliminate their residual dependence on blood pressure. Conversion from CAVI to CAVI_0_ was performed using a Microsoft Excel workbook provided by (Spronck et al., 2019).

#### 2.2.1 CAVI_0_ during changes in body position

Tilting the body creates a hydrostatic pressure gradient causing arterial stiffness to vary with position along the arterial tree, with pressure change being negative above the aortic arch and positive below. The maximum values of hydrostatic pressure acting at the ankle cuff level were calculated as follows:

$$P_{hyd}=h.\rho .g=\sin\left(\alpha \right).L.\rho .g$$

where: P_hyd_ [mmHg] – hydrostatic pressure, h [m] – vertical height difference between the heart and ankle, calculated as the sine of the head-up tilt angle (α) multiplied by the vascular path length L [m], ρ [kg·m^-3^] – blood density, g [m·s^-2^] – gravitational acceleration constant (9.81 m.s^-2^).

Since the hydrostatic pressure gradient affects arterial stiffness and hence pulse wave velocity (PWV) along the arterial pathway during tilting, the CAVI_0_ calculated from PWV and brachial blood pressure measurements using the VaSera device does not account for these hydrostatic effects. To better approximate the mean arterial stiffness of the heart-to-ankle segment during tilting, we recalculated CAVI_0_ (referred to as CAVI_0 ADJ_) using the measured haPWV and adjusted diastolic blood pressure DBP_ADJ_ = DBP_VaSera_ + P_hyd_/2 (Butlin et al., 2015).

The mean of the right and left CAVI_0 ADJ_ values was used for subsequent analyses.

### 2.3 Cardiovascular parameters

A set of non-invasive, continuous recordings was used to assess cardiovascular function, including basic hemodynamic characteristics, in all participants. These included electrocardiography (ECG; CardioFax ECG-9620, Nihon Kohden, Japan), arterial blood pressure (reconstructed brachial pressure derived from finger arterial pressure using the volume-clamp photoplethysmography method; Finometer Pro, FMS, Netherlands; a Height Correction Unit was used to compensate for hydrostatic pressure changes during head-up tilt test), and impedance cardiography (ICG; CardioScreen 2000; Medis, Germany). All these measurements were continuously recorded throughout the entire study protocol. Participants were instructed to remain still and refrain from speaking during the measurement. During preprocessing, signals from the three devices were synchronized using cross-correlation of R–R intervals from ECG and ICG and pulse intervals from the Finometer.

R–R intervals were derived from the electrocardiogram as the time intervals between consecutive R-wave peaks. Heart rate (HR) was then calculated by dividing 60 by the R–R interval in seconds. Systolic and diastolic blood pressure values were defined as the maximal and minimal values of the recorded arterial pressure waveforms, respectively. Detection of R waves, as well as quantification of SBP and DBP, was performed using the available ECG and Blood Pressure analysis modules implemented in LabChart. Mean blood pressure (MBP) was determined as the integral under the curve of the reconstructed brachial pressure waveform, continuously and non-invasively measured using the Finometer device. Median values of impedance cardiography measures were used to reduce the impact of rare artifacts in the measurements. Systemic vascular resistance (SVR), an index of overall vasomotion state, was calculated using the following formula:

$$SVR=80\;*\;\left(\text{median }MBP/\text{median }CO\right)$$

where: SVR [dyns·cm^-5^] – systemic vascular resistance, MBP [mmHg] – mean arterial pressure derived from the reconstructed blood pressure curve, CO [L·min^-1^] – cardiac output estimated using ICG, the constant 80 serves as a conversion term to ensure unit consistency. Central venous pressure was assumed negligible in this calculation. All cardiovascular parameters were derived from mean values computed across 300 consecutive cardiac cycles.

### 2.4 Statistical analysis

The normality of the data distribution was assessed using the Shapiro-Wilk test. Variables are presented as mean ± SD for normally distributed data or median [interquartile range] for non-normally distributed data.

Repeated-measures analysis of variance (ANOVA) was employed to evaluate differences in CAVI_0 ADJ_ and other monitored cardiovascular parameters across various body positions, with *post hoc* comparisons adjusted using the Bonferroni correction for variables meeting assumptions of data normality at all measurement time points. For variables in which at least one measurement violated parametric tests assumptions, the nonparametric Friedman test with pairwise *post hoc* comparisons was applied. Paired *t*-test for normally distributed variables or Wilcoxon test for non-normally distributed variables were used to compare parameters between baseline and MA task.

Correlation analyses pooled across all tilt angles (3 positions in 23 participants resulted in 69 measurements) were performed to examine associations between position-induced changes in the arterial stiffness index CAVI_0_ (%ΔCAVI_0__ADJ_) and corresponding changes in hemodynamic parameters. Spearman’s rank correlation was applied because %ΔCAVI_0__ADJ_ was not normally distributed.

A p-value <0.05 was considered as statistically significant. Statistical analysis was performed using the SYSTAT software version 13.1 (Systat Software, Inc., California, United States).

## 3 Results

In our study group, supine CAVI_0_ showed no correlation with age (ρ = 0.021, p = 0.924), anthropometric measures (height: R = 0.334, p = 0.120; weight: R = 0.199, p = 0.363; BMI: ρ = 0.305, p = 0.157, fat percentage: ρ = −0.313, p = 0.146, fat mass: ρ = −0.173, p = 0.430, skeletal muscle mass: ρ = 0.278, p = 0.199, VFA (Visceral Fat Area): ρ = 0.164, p = 0.455, WHR (Waist-to-Hip Ratio): R = 0.215, p = 0.336), or basic cardiovascular parameters in the supine position, including HR (R = −0.140, p = 0.525), SBP (R = 0.082, p = 0.710), DBP (R = −0.113, p = 0.608), and MBP (R = −0.001, p = 0.997). Due to the limited variability in the anthropometric measures within our cohort, we did not perform correlation analyses between these variables and the magnitude of the CAVI_0_ response to the orthostatic stimulus.

### 3.1 Changes in body position

**FIGURE 1:**
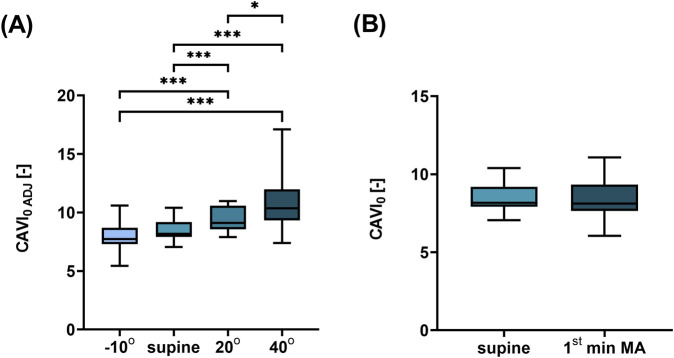
Box plots illustrating: **(A)** changes of CAVI_0 ADJ_ with different body positions (10° head down tilt (HDT), 20° head up tilt (HUT), and 40° HUT); **(B)** comparison of CAVI_0_ between supine rest and first minute of mental arithmetic (MA) task; the box plots report minimum and maximum values (whiskers) and (25, 50, 75)th percentiles (box with central line). Statistical significance is indicated as follows: *p < 0.05; ***p < 0.001.

The main effects of body position changes are presented in Figure 1A; Table 2. Estimated hydrostatic pressures at the ankle were - 8.5 ± 1.1 mmHg (10° HDT), 36.4 ± 2.1 mmHg (20° HUT), and 68.4 ± 3.9 mmHg (40° HUT).

**TABLE 2:** Hemodynamic parameters alterations induced by changes in body position.

Parameters	10° HDT	Supine	20° HUT	40° HUT	*p* (trend)	*p* Supine vs 10° HDT	*p* Supine vs 20° HUT	*p* Supine vs 40° HUT	*p* 10° HDT vs 20° HUT	*p* 10° HDT vs 40° HUT	*p* 20° HUT vs 40° HUT
CAVI_0 ADJ_ [-]	7.96 ± 1.12	8.39 ± 0.86	*9.10 ± [8.57–10.58]*	*10.35 [9.45–11.92]*	*< 0.001*	*ns*	*< 0.001*	*< 0.001*	*< 0.001*	*< 0.001*	*0.016*
haPWV [m·s^-1^]	5.75 ± 0.49	6.13 ± 0.50	6.84 ± 0.49	7.60 ± 0.68	< 0.001	0.002	< 0.001	< 0.001	< 0.001	< 0.001	< 0.001
HR [bpm]	64.7 ± 9.9	66.4 ± 9.9	70.4 ± 9.0	79.5 ± 8.2	< 0.001	ns	0.010	< 0.001	0.002	< 0.001	< 0.001
MBP [mmHg]	90.8 ± 7.3	90.4 ± 6.7	92.6 ± 8.6	91.7 ± 7.5	ns	*ns*	*ns*	*ns*	*ns*	*ns*	*ns*
SVR [dyn⋅sec⋅cm^-5^]	1,112.5 ± 213.4	1,081.4 ± 204.2	1,202.6 ± 223.4	1,239.0 ± 227.8	< 0.001	ns	0.001	< 0.001	0.015	< 0.001	ns

CAVI_0 ADJ_, corrected cardio-ankle vascular index adjusted for the blood pressure acting along the measured arterial segment, incorporating the contribution of hydrostatic pressure; haPWV, heart-ankle pulse wave velocity; HR, heart rate; MBP–brachial mean blood pressure; SVR, systemic vascular resistance; data are presented as mean ± SD (normally distributed variables, ANOVA) or *median [interquartile range]* (non-normally distributed variables, Friedman test); “ns” – non-significant.

Heart-to-ankle pulse wave velocity (haPWV), which is not corrected for the influence of arterial blood pressure, also increased significantly with increasing tilt angle (p for trend <0.001), and all pairwise comparisons reached statistical significance (all p ≤ 0.002). Importantly, despite adjustment for diastolic blood pressure in the examined arterial segment, CAVI_0 ADJ_ remained significantly affected by body position (p for trend <0.001). Post hoc analysis demonstrated no difference between 10° HDT and the supine position; however, CAVI_0__ADJ_ was significantly higher at both 20° and 40° HUT compared with supine and 10° HDT. Furthermore, a significant increase was observed between 20° and 40° HUT, indicating a stepwise elevation with increasing orthostatic stress.

Heart rate rose progressively with increasing tilt angles. Significant increases were observed between supine and upright positions and between head-down and upright tilt, with an additional increase at 40° compared with 20° head-up tilt. Brachial mean blood pressure (MBP) remained unchanged across positions. Systemic vascular resistance (SVR) also increased with upright tilt (p < 0.001). Pairwise comparisons confirmed higher vascular resistance during both upright positions compared with supine and head-down tilt, while SVR did not differ between the 20° and 40° upright positions.

**FIGURE 2:**
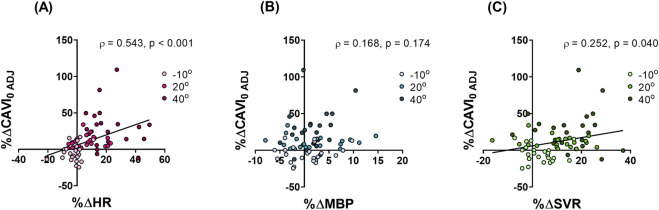
Correlation between percentage change in CAVI_0 ADJ_ (corrected cardio-ankle vascular index adjusted for the blood pressure acting along the measured arterial segment, incorporating the contribution of hydrostatic pressure) and **(A)** percentage changes in heart rate (%ΔHR), **(B)** brachial mean blood pressure (%ΔMBP), and **(C)** systemic vascular resistance (%ΔSVR); pooled across all tilting angles (-10°, 20°, and 40°); %Δ = (variable in tilt–variable in supine)/variable in supine; linear regression lines are shown for illustrative purposes; correlation coefficients (ρ) and corresponding p-values are indicated for each relationship; sample size = 69 (23 participants x three tilt angles).

Correlation analysis (Figure 2) showed a significant positive correlation between percentage change in CAVI_0__ADJ_ and percentage change in heart rate (p < 0.001). No correlations were observed between a change in CAVI_0__ADJ_ and a change in brachial mean blood pressure (p = 0.174). A weak but statistically significant association was found between percentage change in SVR and CAVI_0__ADJ_ (p = 0.040).

### 3.2 Mental arithmetic task

The main effect of mental arithmetic task on CAVI_0_ and cardiovascular parameters is presented in Table 3. During MA task most hemodynamic parameters increased compared with basal conditions. haPWV, brachial MBP, and HR increased significantly. Systemic vascular resistance (SVR) also showed a modest but significant increase. In contrast, CAVI_0_ did not change significantly (Figure 1B).

**TABLE 3:** Comparison of hemodynamic parameters between supine rest and MA task.

Parameters	Supine rest	MA (1^st^ min)	*p*
CAVI_0_ [-]	8.39 ± 0.86	8.47 ± 1.29	ns
haPWV [m⋅s^-1^]	6.13 ± 0.50	6.50 ± 0.55	**0.001**
HR [bpm]	66.4 ± 9.9	77.4 ± 14.6	**< 0.001**
MBP [mmHg]	*90.6 [86.6–93.9]*	*103.6 [92.2–107.6]*	***< 0.001***
SVR [dyn⋅sec⋅cm^-5^]	1,081.4 ± 204.2	1,126.4 ± 198.3	**0.017**

CAVI_0_, corrected cardio-ankle vascular index; haPWV, heart-ankle pulse wave velocity; HR, heart rate; MBP, brachial mean blood pressure; SVR, systemic vascular resistance; MA, mental arithmetic task; data are presented as mean ± SD (normally distributed variables, t-test) or *median [interquartile range]* (non-normally distributed variables, Wilcoxon signed-rank test); “ns” – non-significant.

## 4 Discussion

Each cardiovascular parameter–a node in the cardiovascular network–should be considered in the context of its interconnections with the surrounding nodes. Therefore, an interpretation of the single measure without having information on its potential confounders should be considered as oversimplified. Although arterial stiffness was regarded as the mechanical characteristics of the arterial tree undergoing mostly long-term changes associated with the process of aging or atherosclerosis progression (Lee and Oh, 2010), this characteristic has its own short-term dynamics (Svec et al., 2021; Nardone et al., 2020).

The main goal of our study was to examine the dynamics of CAVI_0_ (theoretically independent of transient blood pressure changes, thereby reflecting intrinsic arterial stiffness) and its potential confounders across varying physiological states, including changes in body position and mental stress.

The main findings of our study are: i) CAVI_0__ADJ_, designed to approximate a pressure-independent index of arterial stiffness along the heart–ankle arterial segment (accounting also for the hydrostatic pressure effect), demonstrated systematic modulation with changes in body position during head-up tilt (HUT). This observation indicates that, even after correction for hydrostatic effects, CAVI_0__ADJ_ remains sensitive to posture-related cardiovascular alterations. Furthermore, magnitude of CAVI_0 ADJ_ alterations correlated with changes in HR and—to a lesser extent—with changes in SVR. ii) Mental stress elicited significant changes in cardiovascular parameters, including blood pressure, HR, and SVR; however, arterial stiffness as quantified by CAVI_0_ was unaffected.

### 4.1 Changes in body position–effect on arterial stiffness

The most common and recommended body position for measuring arterial stiffness is a supine position. However, in clinical practice, it is not uncommon to permit alternative body positions for patient comfort, particularly in individuals with conditions such as low back pain or those recovering from abdominal surgery (Schroeder et al., 2017; Karaki et al., 2025). This has prompted several studies to examine the impact of body position on arterial stiffness parameters. Studies employing carotid–femoral pulse wave velocity (cfPWV), the most commonly used method for assessing arterial stiffness, have reported significant increase in cfPWV during HUT (Huijben et al., 2012; Pucci et al., 2020), in the sitting position (Butlin et al., 2018; Karaki et al., 2025), and in Fowler’s position (position in which the head of the bed is elevated to a semi-sitting position) (Schroeder et al., 2017; Alhalimi et al., 2023). Changes in CAVI across different body positions have also been investigated. Zwain et al. (2013) reported a positive correlation between CAVI and the degree of HUT. Schroeder et al. (2017) observed progressively increasing CAVI values with upper body tilting to 45° and 72°. Shimawaki and Yusa (2016) found higher CAVI values in sitting, sitting with knees flexed at 0°, reclining at 50°, and standing positions compared with the supine position. Maliha and Townsend (2017) further demonstrated that CAVI is sensitive to even small positional variations of ±7°. It should be noted that in these studies, CAVI was determined using the VaSera device, which calculates values based on heart-to-ankle PWV and brachial arterial blood pressure, without accounting for hydrostatic pressure effect in non-supine positions.

The majority of authors attribute posture-induced changes in arterial stiffness parameters to the hydrostatic pressure gradient along the assessed arterial segment, which increases the effect of intraarterial pressure on mechanical properties of arterial wall. However, some studies suggest that postural alterations may increase cfPWV and CAVI beyond blood pressure–related effects, including hydrostatic influences (Pucci et al., 2020; Schroeder et al., 2017).

In this study, we used blood pressure independent index of arterial stiffness CAVI_0_, recalculated with blood pressure adjusted to account for hydrostatic effect, thereby representing the arterial stiffness from the heart to the ankle at different head-up tilt (HUT) angles. If arterial stiffness were only dependent on blood pressure, we assume that CAVI_0 ADJ_ would not change with changes in body position. Our findings of gradually increasing CAVI_0 ADJ_ with increasing HUT angle suggest that arterial stiffness may increase intrinsically, independent of concomitant changes in blood pressure, and that additional mechanisms may contribute to the observed responses.

These findings are consistent with our previous study by Svec et al. (2022), which demonstrated an orthostasis-associated shift in the relationship between arterial blood pressure and arterial compliance (AC) – the reciprocal of arterial stiffness, estimated from diastolic blood pressure decay. Svec et al. (2021) and Svec et al. (2022) also observed a reduction in AC normalized to a blood pressure of 120 mmHg (AC_120_), further emphasizing that factors beyond arterial blood pressure play an important role in the short-term changes in arterial stiffness.

Changes in hemodynamic parameters during head-up tilt have been well documented. The transition from a supine to an upright position reduces venous return, leading to a decrease in cardiac output and arterial blood pressure. Orthostatic stress triggers baroreceptor unloading, resulting in vagal withdrawal and increased sympathetic activity. These autonomic adjustments produce an elevation in heart rate and peripheral vasoconstriction, reflected by an increased SVR, helping to maintain mean blood pressure and overall cardiovascular homeostasis (Lee et al., 2017). Evidence suggests that both heart rate and smooth muscle tone may influence arterial stiffness (Nardone et al., 2020).

Firstly, several studies have demonstrated a significant effect of heart rate on arterial stiffness parameters (Sa Cunha et al., 1997; Lantelme et al., 2002; Tan et al., 2016; Logan et al., 2020). Sa Cunha et al. (1997) was among the first to report that an increased heart rate was strongly associated with an elevated pulse-wave velocity, even after adjustment for age and blood pressure in a large cohort of normotensive and hypertensive individuals. Similar findings were reported by Logan et al. (2020), Logan and Kim (2016), who showed that higher resting HR was independently associated with increased arterial stiffness, measured by cfPWV, in 102 normotensive adults aged 21–60 years. Other investigators have examined the effect of HR on arterial stiffness under controlled conditions in patients with implanted pacemakers or cardioverter devices. These studies (Lantelme et al., 2002; Haesler et al., 2004; Tan et al., 2016) consistently demonstrated a strong effect of HR on cfPWV. The physiological mechanisms underlying this association remain incompletely understood. One proposed explanation relates to the viscoelastic properties of the arterial wall. Increased HR shortens the time available for arterial recoil, which may promote functional stiffening of the vessel wall (Lantelme et al., 2002; Tan et al., 2016). Furthermore, an effect of reflected pulse wave should be considered: with an increase in heart rate, the reflected pulse wave from peripheral parts of arterial tree will return earlier during cardiac cycle (Albaladejo et al., 2001) contributing to increased stretching of elastic arteries augmenting the effect of heart rate on arterial stiffness. From a clinical perspective, the magnitude of HR-related changes in cfPWV appears modest, with reported estimates of approximately 0.16–0.20 m.s^-1^ per 10 beats per minute (Tan et al., 2016). While small fluctuations in HR may have limited physiological relevance, larger HR differences could significantly distort arterial stiffness measurements.

As the second factor, sympathetic input to the arterial system represents an additional confounder when estimating arterial stiffness during changes in body position. Head-up tilt increases cardiovascular sympathetic activity, which alters vascular smooth muscle tone in muscular arteries and thereby modifies arterial stiffness independently of blood pressure. This effect is not uniform throughout the arterial tree, as more peripheral, muscular arteries exhibit larger responsiveness to sympathetic stimulation (Nardone et al., 2018; Nardone et al., 2020; Schroeder et al., 2017). Whether sympathetic outflow can modulate the stiffness of predominantly elastic large arteries—which carry greater prognostic relevance than peripheral arteries—remains controversial. Maki-Petaja et al. (2016) using a three-study protocol, reported that in young healthy subjects the autonomic nervous system does not exert a pressure-independent effect on aortic stiffness. In contrast, Nardone et al. (2018) and Holwerda et al. (2019) documented a significant influence of increased sympathetic activity on carotid-femoral pulse wave velocity during lower body negative pressure simulating orthostatic stress without altering arterial blood pressure. Similarly Svec et al. (2021), Svec et al. (2022) observed increased systemic vascular resistance accompanied by elevated arterial stiffness parameter AC_120_ during 45° head-up tilt. In our study, pulse wave velocity for CAVI_0_ calculation was measured from the heart to the ankle, incorporating arterial segments from the ascending aorta to the tibial arteries. Therefore, we assume that changes in body position—particularly at higher tilt angles—may influence measured arterial stiffness through increased sympathetic activation.

Physiological responses to head-up and head-down tilt are not necessarily symmetrical, suggesting the involvement of distinct regulatory mechanisms (Cunningham et al., 1988). To date, only a single study has investigated changes in arterial stiffness during head-down tilt. Cohen et al. (2020) reported that cfPWV remained unchanged during a 45° HDT compared with supine values; however, cfPWV in that study was not adjusted for the concomitant changes in blood pressure. In the present study, a 10° HDT did not produce significant changes in CAVI_0__ADJ_ or in the monitored hemodynamic parameters relative to the supine condition. Physiologically, the modest tilt angle and short duration of exposure likely provided an insufficient orthostatic stimulus to elicit detectable cardiovascular or arterial stiffness responses.

### 4.2 Mental arithmetic task–effect on arterial stiffness

Mental stress activates the sympathetic nervous system, leading to increases in arterial blood pressure and heart rate together with peripheral vasoconstriction (Muller et al., 2013; Shah et al., 2020; Svec et al., 2021). The majority of studies examining the vascular effects of acute mental stress report concomitant increases in arterial stiffness. Boutouyrie et al. (1994) demonstrated a significant reduction in radial arterial compliance calculated at 100 mmHg during mental stress. Similarly, Vlachopoulos et al. (2006) and Vlachopoulos et al. (2009) showed that mental arithmetic, as well as emotionally stressful movie stimulus, acutely increased carotid–femoral PWV, with effects persisting for up to 60 min. Logan et al. (2020) reported a significant rise in cfPWV following the Trier Social Stress Test compared with controls, independent of age, BMI, and systolic blood pressure. Additional studies confirmed increases in regional PWV and CAVI during mental arithmetic, with evidence of segmental heterogeneity in the stiffness response (Gentilin et al., 2023; Matsumura et al., 2019; Kume et al., 2020). Nevertheless, not all indices demonstrate uniform changes, as carotid artery stiffness (common carotid artery elastic modulus, and β-stiffness index) remained unchanged during a Stroop task in the study by Heffernan et al. (2015), highlighting methodological and regional variability.

In contrast to many of these reports, we did not observe a significant change in CAVI_0_ between supine rest and mental arithmetic, despite clear increases in heart rate, blood pressure, and systemic vascular resistance. Importantly, CAVI_0_ was specifically developed to minimize the influence of acute blood pressure fluctuations on arterial stiffness assessment. Whereas conventional PWV measures are inherently pressure-dependent, CAVI_0_ is derived to more closely reflect intrinsic arterial wall properties independent of actual pressure changes. The absence of a significant change in CAVI_0_ in our study, despite marked sympathetic and pressor responses (increased HR, MBP and SVR during MA), therefore supports the conceptual advantage of this index in isolating structural arterial wall stiffness from acute blood pressure–mediated effects. We suggest that increases in arterial stiffness reported in previous studies could be–to some extent–attributable to concomitantly observed increases in arterial blood pressure values.

### 4.3 Factors influencing arterial stiffness during orthostasis and cognitive load–summarization

The results of the first part of our study indicate a significant association between increases in CAVI_0__ADJ_ (%ΔCAVI_0__ADJ_) and increases in HR (%ΔHR) and SVR (%ΔSVR), suggesting a possible contribution of heart rate and systemic vascular resistance to changes in arterial stiffness under different postural conditions. Notably, correlation analysis revealed a stronger relationship between %ΔCAVI_0__ADJ_ and %ΔHR than between %ΔCAVI_0__ADJ_ and %ΔSVR (lower correlation strength between %ΔCAVI_0__ADJ_ and %ΔSVR (Figure 2)). The weaker association between changes in CAVI_0__ADJ_ and SVR may have two possible explanations. First, vasomotion may affect vascular stiffness partially independently of vascular resistance; that is, its effects may differ between large and small arteries. Second, systemic vascular resistance is calculated from mean arterial pressure and cardiac output, both of which are subject to measurement error. This may introduce considerable noise into SVR estimates and thereby weaken the observed correlation between %ΔCAVI_0__ADJ_ and %ΔSVR. Taken together, the relatively weak correlation between a change in CAVI_0__ADJ_ and SVR should be interpreted with caution and may not reflect a direct causal relationship.

Interestingly, although mean blood pressure, heart rate, and vascular resistance all changed significantly during the MA task in a manner consistent with increased arterial stiffness, we did not observe a significant change in CAVI_0_. First, the effect of an increased MBP on arterial stiffness was corrected for during the CAVI_0_ calculation; therefore, any influence of elevated MBP on CAVI_0_ was effectively eliminated. Second, the increase in heart rate of 11 bpm during MA task was relatively modest and was therefore unlikely to have a substantial effect on arterial stiffness. Third, although the change in SVR was statistically significant, its magnitude was very small (approximately 4%). It should be noted that SVR is mostly influenced by peripheral vasoconstriction while arterial stiffness is related to the tone of smooth muscles in larger arteries and thus, we do not have direct information about vascular tone in larger arteries. We assume that smooth muscle contraction throughout the arterial tree is not necessarily generalized. Fourth, extravascular pressure exerted on large arteries may also affect arterial stretch and, consequently, arterial stiffness. During the MA task, alterations in breathing patterns were frequently observed (Grassmann et al., 2016), potentially affecting mean intrapleural pressure. Finally, any change in CAVI_0_ may have been below the detection threshold of the measurement. We therefore suggest that the absence of a significant change in CAVI_0_ may result from the interplay of the factors discussed above.

## 5 Clinical implications

From the clinical point of view our findings indicate, that a) any deviation from the supine position alters arterial stiffness parameters. Therefore, arterial stiffness should be assessed in the supine position to ensure methodological consistency and avoid position-related bias; b) CAVI_0_ is influenced by both heart rate changes and/or vasomotion and this index is not only a measure of structural changes of arterial tree but includes also short-term effects of autonomic nervous system that should be minimized during early atherosclerosis assessment in the preventive medicine; c) the arterial stiffness parameter CAVI_0_ is not influenced by acute blood pressure changes caused by moderate mental stress pointing towards its robustness against blood pressure alterations.

## 6 Study limitation

We acknowledge several limitations of this study. First, the sample size is small, thus we cannot exclude a possibility of type II error and caution is needed when interpreting the data. Second, the study included only young, healthy, Caucasian participants to minimize the influence of atherosclerosis and arteriosclerosis, which may limit generalizability to older adults or individuals with elevated sympathetic activity or hypertension. In a previous study, older individuals (>45 years) had a more pronounced increase in arterial stiffness when the entire body was tilted up using the tilt table test (Pucci et al., 2020). On the other hand, the narrow age range and homogeneous anthropometric profile of the participants reduced the likelihood that these factors confounded the observed results. Third, the sex distribution in our sample was unbalanced (16 males vs. 7 females), which may have influenced the results. Large epidemiological studies show a stronger association between blood pressure and arterial stiffness in older women than in men (Ohldieck et al., 2025), yet evidence in younger populations is limited, with many studies reporting similar blood pressure–stiffness relationships across sexes (Yu et al., 2019). Fourth, arterial path length (L) was estimated from body height using the formula implemented in VaSera device (Takahashi et al., 2019) rather than measured directly, which may introduce bias in the CAVI_0_ calculation. Fifth, we did not measure blood pressure at the level of the ankle but estimated it based on a calculation from the subjects’ height. Sixth, only one mental stress task was used. Although the arithmetic test is validated and widely used, it may not elicit high-level stress, and real-world stressors could have a stronger impact on arterial characteristics (Vlachopoulos et al., 2006). In our study, the task induced only mild to moderate subjective stress level (results not presented), and higher levels of acute psychological stress might provoke different patterns of autonomic response and arterial stiffness. Finally, each participant was studied only once, so the reproducibility of the results could not be determined.

## 7 Conclusion

In conclusion, our study demonstrates that the arterial stiffness parameter CAVI_0_ remains unaffected by acute blood pressure changes induced by moderate mental stress. In contrast, during head-up tilt, arterial stiffness is modulated not only by pressure-dependent mechanical effects related to hydrostatic gradients, but also by heart rate and vasomotion. The influence of these important confounders should be considered when interpreting arterial stiffness indices as early indicators of atherosclerotic process progression.

## Data Availability

The raw data supporting the conclusions of this article will be made available by the authors, without undue reservation.
